# Rare indication for DMEK surgery: Treatment of corneal decompensation in patients with radial keratotomy and multifocal lens

**DOI:** 10.22336/rjo.2026.64

**Published:** 2026

**Authors:** Marina Aguilar-González, Mª José Roig-Revert, Cristina Peris-Martínez

**Affiliations:** 1 Unit of Cornea and Anterior Eye Diseases, Hospital de Manises, Manises, Valencia, Spain; 2 Unit of Cornea and Anterior Eye Diseases, Hospital Universitario Doctor Peset, Valencia, Spain; 3 Aviñó Peris EyeClinic, Valencia, Spain; 4 Department of Surgery, Ophthalmology, Universitat de Valencia, Valencia, Spain; 5 Unit of Cornea and Anterior Eye Diseases, Fundación de Oftalmología Médica de la Comunidad Valenciana (FOM), Valencia, Spain

**Keywords:** DMEK, radial keratotomy, multifocal lens, corneal transplant, corneal topography, AS-OCT = Anterior segment optical coherence tomography, BCVA = Best corrected visual acuity, D = diopters, DMEK = Descemet’s Membrane Endothelial Keratoplasty, DSAEK = Descemet’s stripping automated endothelial keratoplasty, IOL = Intraocular lens, K1 = Flat keratometry, K2 = Curved keratometry, Kmax = Maximum keratometry, LE = Left eye, PKP = Penetrating keratoplasty, RE = Right eye, RK = Radial keratotomy, SL = Slit lamp

## Abstract

**Introduction:**

Radial keratotomy has been associated with endothelial cell loss. Although this complication has traditionally been treated with Descemet’s stripping automated endothelial keratoplasty (DSAEK) or penetrating keratoplasty (PKP), DMEK surgery has recently been successfully performed in some cases.

**Case report:**

In our case, DMEK surgery resulted in a decrease in apex pachymetry of 189, a decrease in Kmax of 3 D, a decrease in sphere of 5.75 D, and a decrease in keratometric astigmatism and refractive astigmatism of 0.60 and 1.50, respectively.

**Discussion:**

Few cases have been published on this subject, and our case adds new information to the limited evidence available.

**Conclusion:**

The data from this clinical case suggest that DMEK surgery can be considered for the treatment of corneal decompensation in eyes that have undergone RK surgery, even in the case of significant topographical abnormalities, as it is associated with a decrease in astigmatism and hyperopia, due to corneal remodeling, which makes it more regular, approaching refractive neutrality.

## Introduction

Descemet’s Membrane Endothelial Keratoplasty (DMEK) has revolutionized corneal transplantation since the technique was described by Dr. Gerrit R.J. Melles in 2006, allowing selective replacement of the Descemet’s membrane and corneal endothelium, eliminating the stromal interface present in previous posterior lamellar keratoplasty techniques such as Descemet’s Stripping Automated Endothelial Keratoplasty (DSAEK) [1]. It has also shown numerous advantages over penetrating keratoplasty (PKP), including faster recovery, better visual outcomes, smaller incisions, lower rejection rates, and less compromise of eyeball integrity. DMEK surgery has become a standardized procedure for Fuchs’ endothelial dystrophy and simple bullous keratopathy; however, eyes with more complex diseases present unique challenges for DMEK [2].

Radial keratotomy (RK) is a refractive surgery used to correct myopia but is now rarely used because of multiple complications, including endothelial cell loss. Corneal alterations in patients with a history of RK have previously been treated with PKP or DSAEK [3], and more recently, DMEK surgery has been performed successfully in some cases [2,4,5]. We present the case of a patient with a history of RK surgery and cataract surgery with a multifocal intraocular lens (IOL) who came to our center with vision loss due to corneal decompensation and was treated with DMEK surgery with anatomical and visual success.

## Case report

A 61-year-old male patient with a history of RK surgery in both eyes (for 5 dioptres of previous myopia) was referred to our center for corneal transplantation due to corneal decompensation following uncomplicated cataract surgery in the right eye (RE) with a multifocal IOL.

The patient had a best-corrected visual acuity (BCVA) of 0.2+/-: 2 with +3.75 (-2.0 x 50º) in the RE and 0.9+/-: -2 with -3.25 (-0.5 x 90º) in the left eye (LE) and intraocular pressure of 8 and 16 mmHg in the RE and LE, respectively. Slit lamp (SL) examination and anterior segment optical coherence tomography (AS-OCT) revealed corneal oedema with folds, radial keratotomy incisions, and a multifocal IOL in the RE (**Fig. 1A-C**) and a clear cornea with RK incisions and incipient phaco-sclerosis in the LE. The fundus of both eyes was normal. Corneal apex pachymetry on AS-OCT was 741 µm in the right eye (**Fig. 2A**) and 555 µm in the left eye. Endothelial cell count was impracticable (0 cells) in the right eye and 2683 cells in the left eye. In the topography of the RE, the cylinder (CYL) was +1.5 dioptres (D), the flat keratometry (K1) was 31.2 D, the curved keratometry (K2) was 33.2 D, and the maximum keratometry (Kmax) was 47.5 D (**Fig. 2B**).

**Fig. 1:**
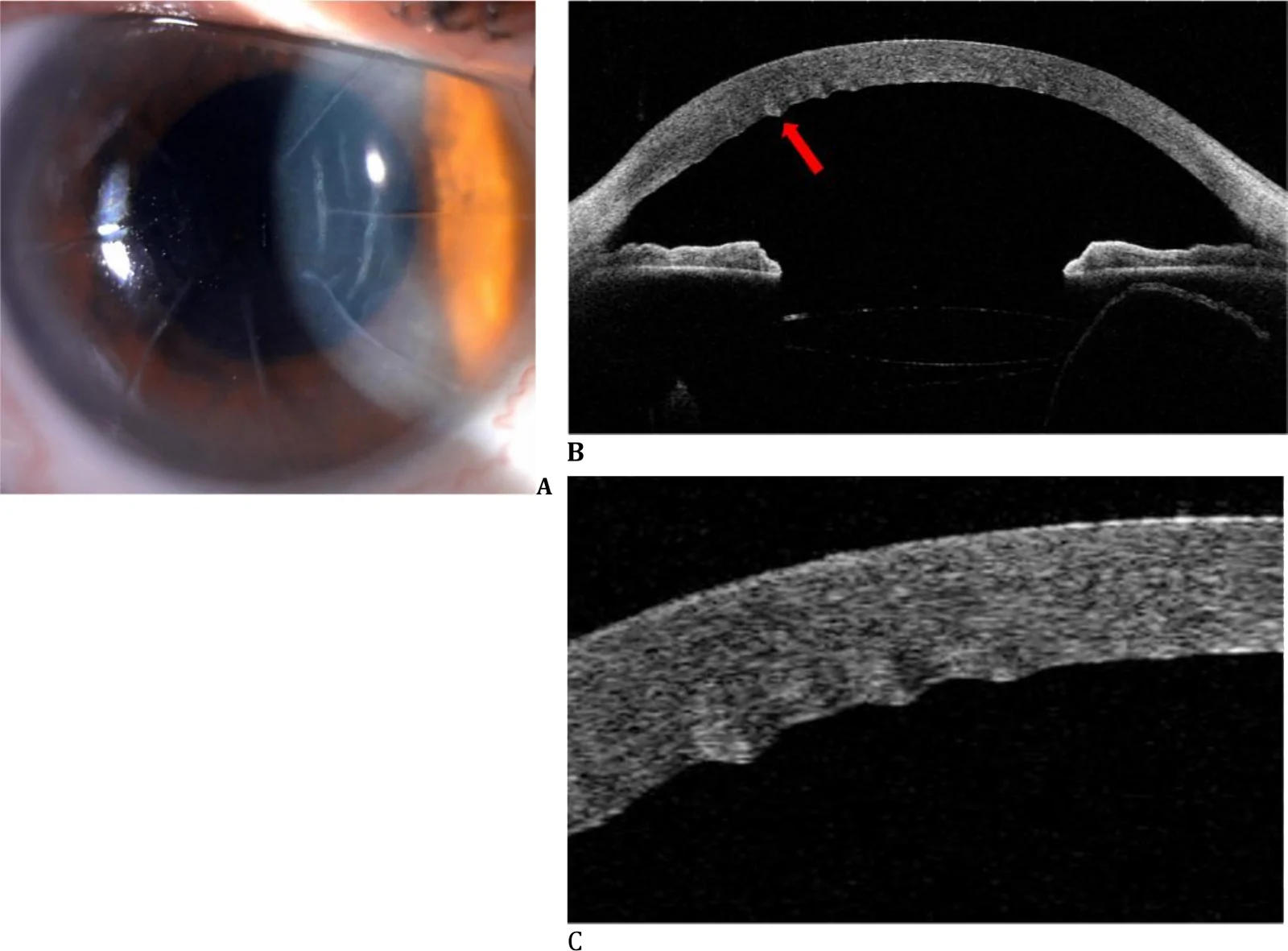
Preoperative image of the RE. **A**) SL photograph showing RK incisions and corneal oedema with Descemet’s folds. **B**) AS-OCT image showing corneal oedema with endothelial folds (indicated by the red arrow). **C**) Enlarged image of the endothelial folds indicated by the arrow in the AS-OCT

**Fig. 2:**
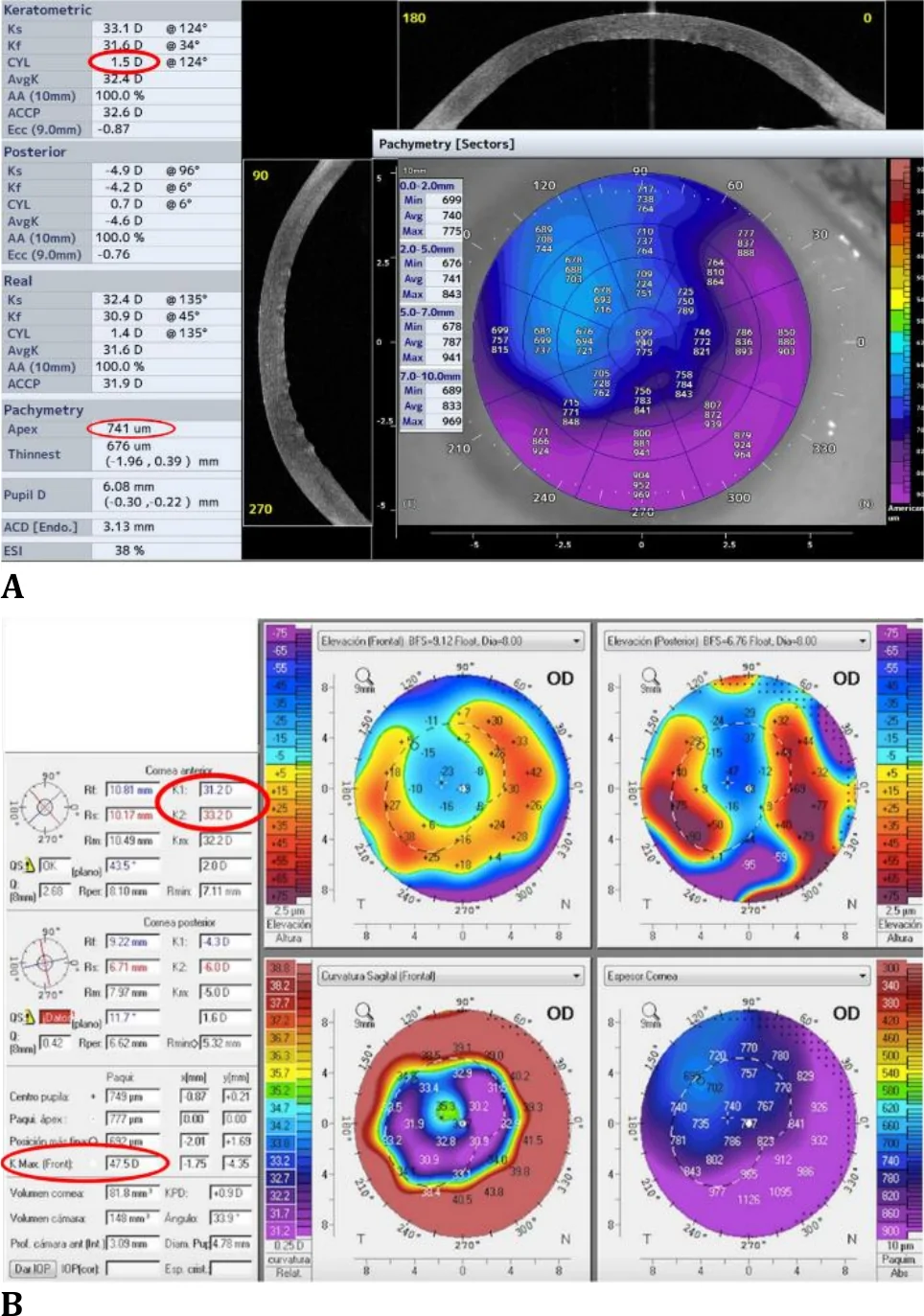
Preoperative data of the RE. **A**) Numerical data from the AS-OCT showing a keratometric cylinder of +1.5 D (124º) and an apex pachymetry of 741 µm (marked with red ovals) before DMEK surgery. **B**) Numerical data from Pentacam showing a K1 of 31.2 D, K2 of 33.2 D, and Kmax of 47.5 D (marked with red ovals) before DMEK surgery

Given these findings, during the first visit, the patient was treated with a subconjunctival injection into the trigone and home treatment with topical 1% prednisolone acetate on a tapering schedule (1 drop every 2 hours for 1 week, then every 6 hours for 2 weeks, every 8 hours for 2 weeks, every 12 hours for 2 weeks, and every 24 hours until the next visit), 5% sodium chloride every 8 hours, and 0.5% topical timolol every 12 hours. The patient was also informed of the possibility of DMEK surgery if there was no improvement with medical treatment.

After one month of topical treatment, BCVA was 0.3, and SL examination showed persistent corneal oedema with folds in the RE; therefore, standardized DMEK surgery was performed using the usual technique with an 8.25 mm endothelial graft in the RE.

On the first day after DMEK surgery in the RE, intraocular pressure was 19, and on examination with SL and AS-OCT, the cornea was clear, the DMEK was in place, the 20% SF6 gas bubble persisted, occupying ½ of the anterior chamber, and the multifocal IOL remained clear in the sac. One week after DMEK surgery, BCVA was 0.3, intraocular pressure was 18, and examination with SL showed a clear cornea. The DMEK remained in place without gas (**Fig. 3A**). AS-OCT showed that pachymetry was 675 µm (**Fig. 3B**). Given the discrepancy between the excellent physical examination and the low BCVA, a fundus examination and macular OCT were performed, revealing the presence of cystic macular oedema in the right eye (**Fig. 3C**). Ketorolac 5% eye drops were added, 1 drop every 8 hours for 1 month. After one month, BCVA in the right eye was 0.8 with -0.5 (-1.0 x 150º) and in the left eye was 0.8 with -5.0 (-1.5 x 145º). On SL examination, the DMEK looked excellent (**Fig. 4A**), RK and multifocal IOL remained stable, AS-OCT showed a pachymetry of 540 µm (**Fig. 4B**), the endothelial cell count was 2296, and the fundus was normal with resolution of cystic macular oedema in the RE (**Fig. 4C**). Shortly afterward, cataract surgery with a monofocal IOL was scheduled for the left eye due to the existing anisometropia, with excellent results.

**Fig. 3:**
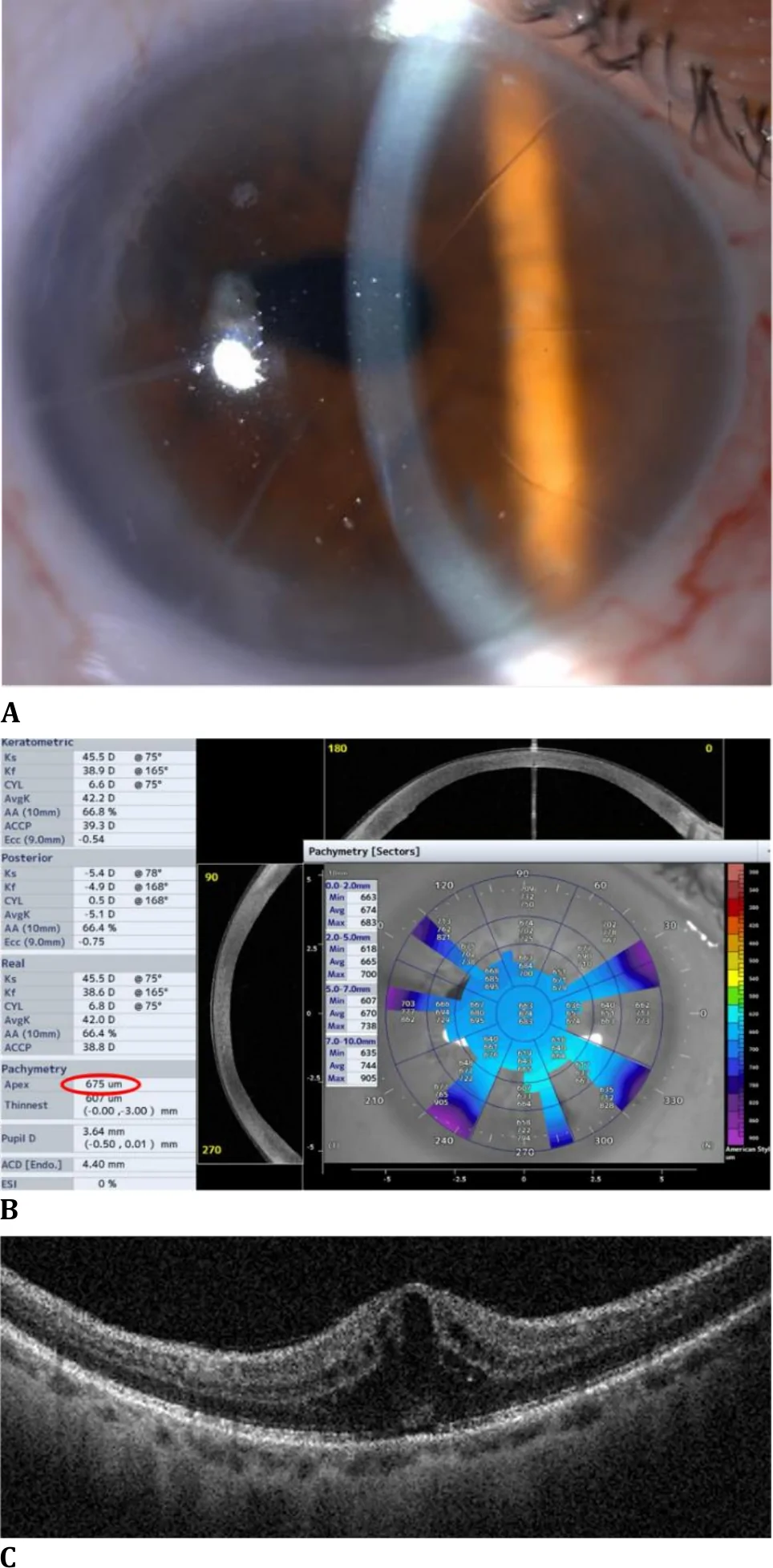
Images one week after DMEK surgery in the RE. **A**) SL photograph showing a clear, transparent cornea with an adhered endothelial graft. **B**) AS-OCT image showing the adhered graft and a decrease in apex pachymetry to 675 µm one week after surgery. **C**) OCT image of the macula showing cystoid macular oedema

**Fig. 4:**
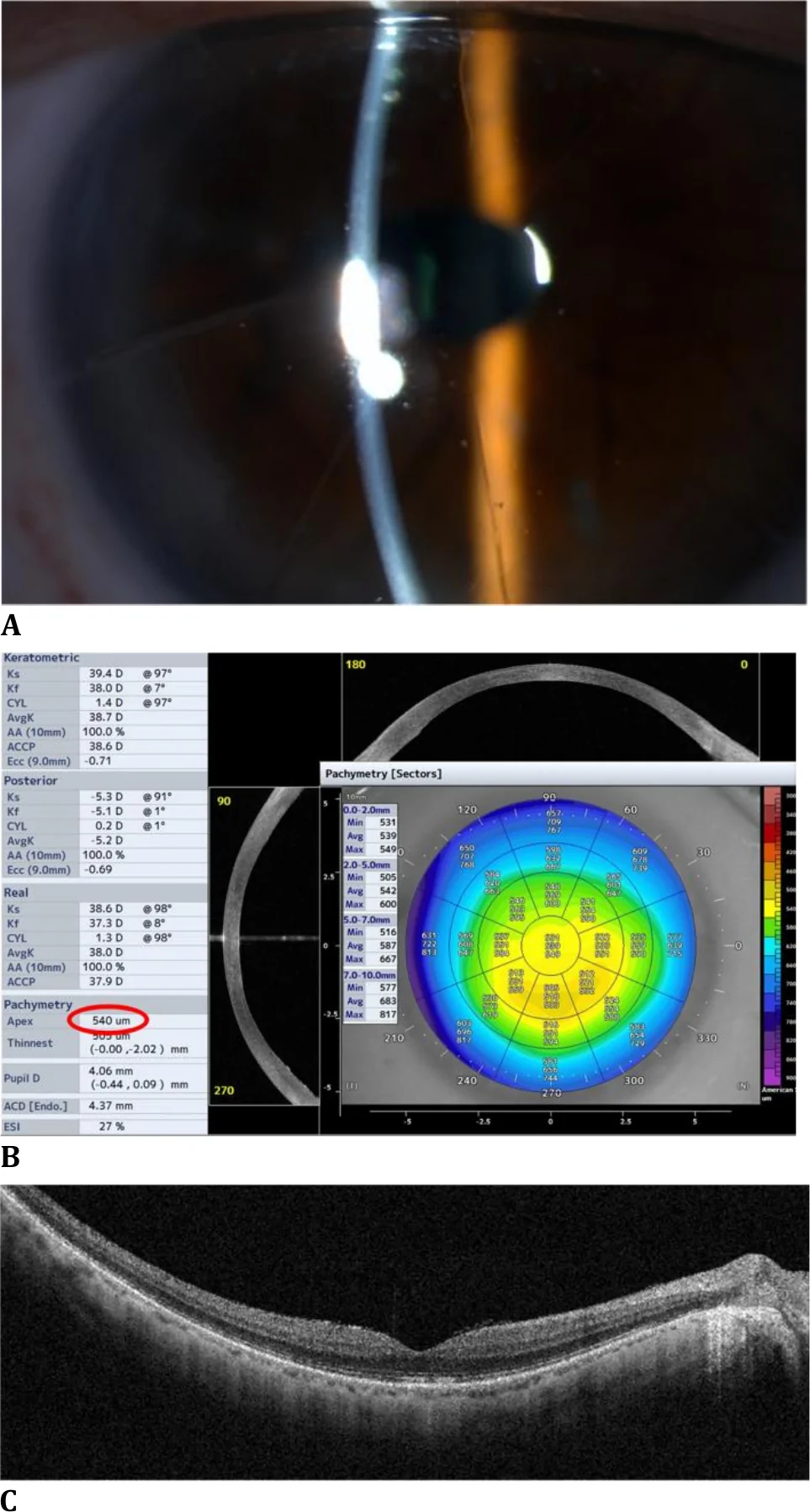
Images one month after treatment of cystoid macular oedema in the right eye. **A**) SL photograph showing a clear, transparent cornea with an adhered endothelial graft. **B**) AS-OCT image showing the adhered graft and a decrease in apex pachymetry to 540 µm one month and one week after surgery. **C**) OCT image of the macula showing preserved foveal profile with complete resolution of cystoid macular oedema

At 2 years, BCVA was 1 with -1.5 (-0.5 x 80°) addition +2.5 in the RE and 1 with +1.0 (-0.75 x 155°) addition +2.5 in the LE. On SL and AS-OCT examination, the endothelial graft, KR incisions, and multifocal IOL remained unchanged in the RE (**Fig. 5A-C**), and the monofocal IOL and cornea with RK incisions remained unchanged in the LE. Fundus examination of both eyes was normal, with no recurrence of cystoid macular oedema in the RE (**Fig. 5D**). Final pachymetry was 552 µm (**Fig. 6A**), and the endothelial cell count was 2074 cells in the RE. In the topography, the final CYL was -0.9 D, K1 was 38.3 D, K2 was 40 D, and Kmax was 44.5 D (**Fig. 6B**). **Table 1** shows the variation in refractive and topographic values before and after surgery (CYL: from +1.5 D to -0.90 D with a change in the steep meridian axis; apex pachymetry: from 741 µm to 552 µm; K1: from 31.2 D to 38.3 D; K2: from 33.2 D to 40 D; Kmax: from 47.5 D to 44.5 D).

**Fig. 5:**
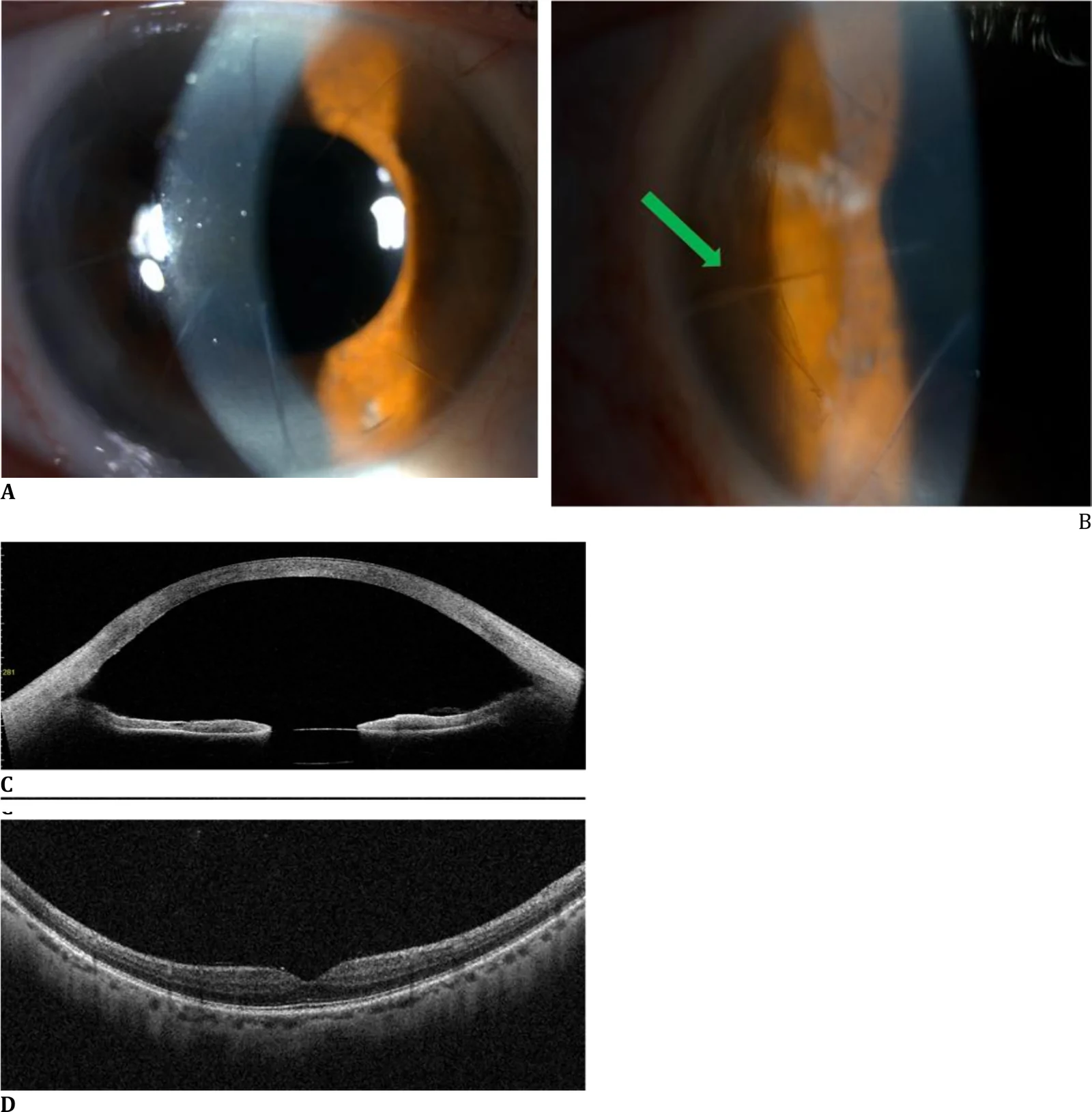
Images two years after DMEK surgery in the RE. **A**) SL photograph showing the transparent cornea. **B**) Detail of SL photograph showing the edge of the endothelial graft and desmetorhexis (green arrow). **C**) AS-OCT image showing the normal morphology of the cornea. **D**) OCT image of the posterior pole showing the preserved foveal profile without cystoid macular oedema

**Fig. 6:**
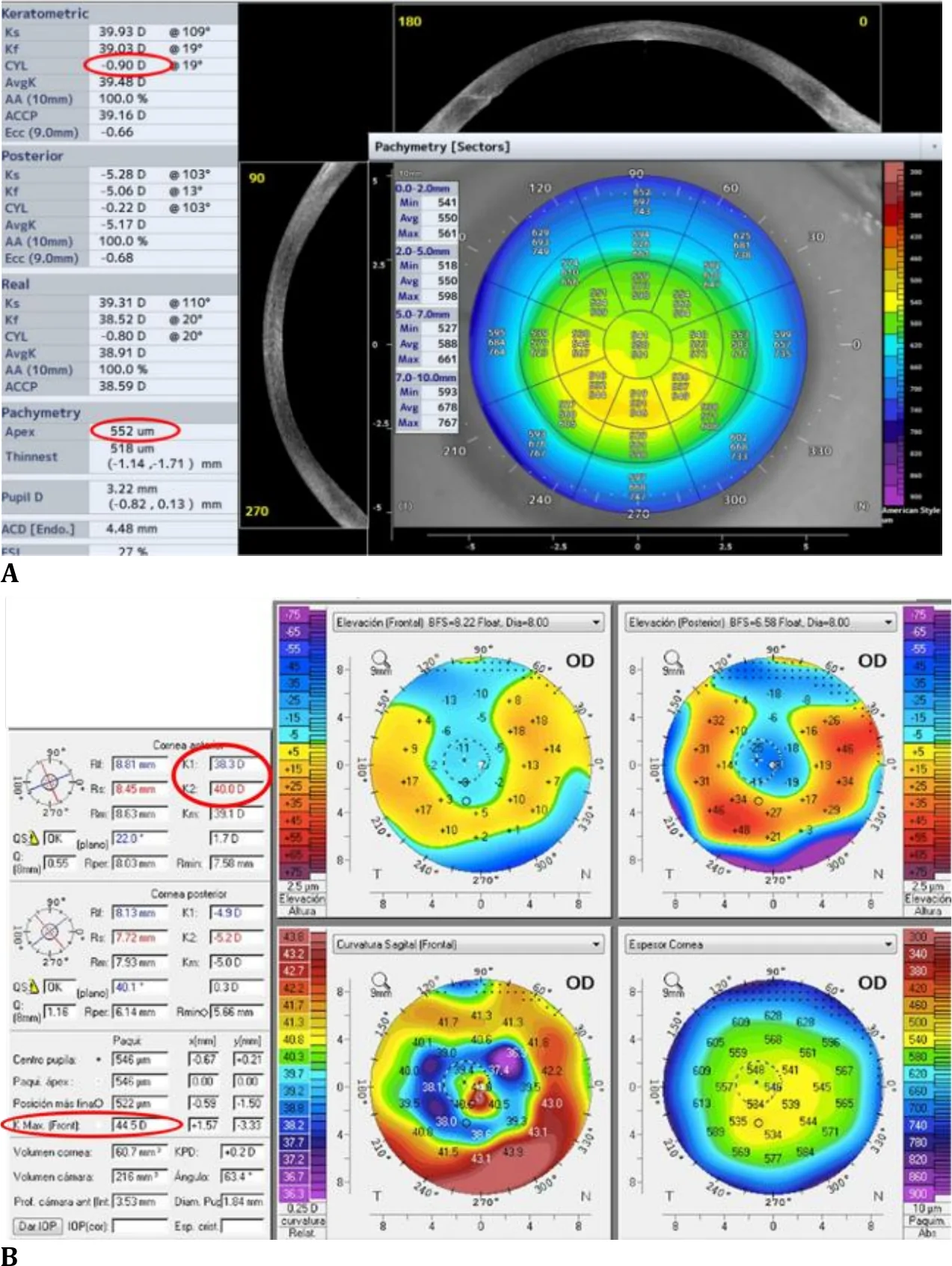
Postoperative data in the RE. **A**) Numerical data from the AS-OCT showing a keratometric cylinder of -0.9 D (19º) and an apex pachymetry of 552 µm (marked with red ovals) after DMEK surgery. **B**) Numerical data from Pentacam showing a K1 of 38.3 D, K2 of 40 D, and Kmax of 44.5 D (marked with red ovals) after DMEK surgery

**Table 1:** Variation in refractive and topographic values before and after surgery

	Preoperative	Postoperative	Difference
Keratometric cylinder (D)	+1,5 (124º)	-0,9 (19º)	-0,60
Pachymetry apex (µm)	741	552	189
K1 (D)	31,2	38,3	-7,1
K2 (D)	33,2	40	-6,8
Kmax (D)	47,5	44,5	+3
Sphere (D)	+3,75	-1,5	+5,25
Refractive cylinder (D)	-2 (50º)	-0,5 (80º)	-1,5

## Discussion

DMEK is considered a refractive surgery with nearly neutral effects because it can minimally affect the anterior corneal curvature of the recipient eye. However, corneas with concomitant stromal abnormalities (e.g., keratoconus) can undergo significant surface remodeling after DMEK, so this could also occur in eyes with RK [4]. In addition, most eye surgeries (cataract surgery, PKP, and DSAEK) have worse outcomes in patients with previous RK than in eyes with isolated endothelial disorders, suggesting this may also occur with DMEK [4]. However, contrary to expectations, published clinical results for DMEK in RK are similar to those in eyes without previous RK. Furthermore, published cases show that after DMEK in eyes with RK, the corneas become more regular, with decreased irregular astigmatism, decreased hyperopia, and an average decrease in Kmax [2,4], as in our patient. Specifically, previous publications have reported a reduction in keratometric astigmatism of 3 D² and a reduction in Kmax of 4.2 D³. In our case, in line with published evidence, Kmax decreased by 3 D, hyperopia decreased by +5.25 D, and the refractive cylinder decreased from 2.00 D preoperatively to 0.50 D postoperatively (Δ −1.50 D). In comparison, keratometric cylinder magnitude decreased from 1.50 D to 0.90 D (Δ −0.60 D) with axis shift.

These findings suggest that DMEK surgery in patients who have previously undergone RK surgery not only avoids hyperopic jump or irregular astigmatism, but also reduces hyperopia, keratometric and refractive astigmatism, and Kmax after surgery, generally leading to refractive neutrality.

The data from this clinical case suggest that DMEK surgery can be considered for the treatment of corneal decompensation in eyes that have undergone RK surgery, even in the case of significant topographical abnormalities, replacing PKP (due to its greater technical difficulty in these eyes and unpredictable visual outcome) and DSAEK surgery (which induces a hyperopic change of +1.25 to +1.50 D), as DMEK surgery has not been associated with the induction of additional irregular astigmatism or such hyperopic displacement. On the contrary, it is associated with a decrease in both due to corneal remodeling, which makes it more regular.

Finally, DMEK surgery can be difficult if the anterior chamber or cornea are altered. Some possible difficulties during DMEK surgery in eyes that have previously undergone RK are: corneal scarring (which affects the ability to remove the Descemet’s membrane); difficulty in reducing the depth of the anterior chamber (as patients are likely to be highly myopic); and, finally, that the RK incisions themselves weaken the structure of the cornea, causing inversion of the corneal curvature during puncture and a decrease in the depth of the central chamber with an increase in the depth of the peripheral AC, making it difficult to move the endothelial graft. One method described to facilitate graft manipulation during surgery is using a pilot bubble and external pressure on the sclera with a spatula to preserve corneal convexity and allow DMEK graft deployment and gas injection.

## Conclusion

In conclusion, the data presented suggest that DMEK can be considered in eyes with previous RK, even in cases of significant topographical abnormalities, replacing PKP and DSAEK, since DMEK has not been associated with hypermetropic increase or additional irregular astigmatism and has been associated with a decrease in astigmatism, hyperopia, and Kmax, providing excellent anatomical and visual results.
